# Dry friction of microstructured polymer surfaces inspired by snake skin

**DOI:** 10.3762/bjnano.5.122

**Published:** 2014-07-21

**Authors:** Martina J Baum, Lars Heepe, Elena Fadeeva, Stanislav N Gorb

**Affiliations:** 1 Functional Morphology and Biomechanics, Zoological Institute, Kiel University, Am Botanischen Garten 1–9, Kiel 24098, Germany; 2 Laser Zentrum Hannover e.V. (LZH), Hollerithallee 8, Hannover 30419, Germany

**Keywords:** biomimetics, dry friction, microstructure, polymer, snake skin

## Abstract

The microstructure investigated in this study was inspired by the anisotropic microornamentation of scales from the ventral body side of the California King Snake (*Lampropeltis getula californiae*). Frictional properties of snake-inspired microstructured polymer surface (SIMPS) made of epoxy resin were characterised in contact with a smooth glass ball by a microtribometer in two perpendicular directions. The SIMPS exhibited a considerable frictional anisotropy: Frictional coefficients measured along the microstructure were about 33% lower than those measured in the opposite direction. Frictional coefficients were compared to those obtained on other types of surface microstructure: (i) smooth ones, (ii) rough ones, and (iii) ones with periodic groove-like microstructures of different dimensions. The results demonstrate the existence of a common pattern of interaction between two general effects that influence friction: (1) molecular interaction depending on real contact area and (2) the mechanical interlocking of both contacting surfaces. The strongest reduction of the frictional coefficient, compared to the smooth reference surface, was observed at a medium range of surface structure dimensions suggesting a trade-off between these two effects.

## Introduction

Owing to the lack of extremities, the ventral body side of snakes is in almost continuous contact with the substrate. In spite of this, snakes are one of the most successful animal groups in occupying niches on all continents, except for Antarctica [1–3]. From a tribology point of view, their ventral skin surface has to fulfil two opposite functions: (1) to support body propulsion during locomotion by generating high friction in contact with the substrate and (2) to reduce skin material abrasion by generating low friction in forward sliding along the substrate [4]. Anisotropic frictional properties of the snake skin were previously shown by several tribological studies using various techniques at the macro scale [5–9], meso scale [10], and nano scale [11]. These properties must be kept up over a longer period of time until new skin is moulted.

Frictional properties of snake skin in contact with a solid partner depend on (i) the surface energy, (ii) material properties, and (iii) surface topography of the tribo-pair [12–13]. The surface energy of snake skin has been mostly assumed according to the chemical analysis of the skin material [14–18]. Only Lillywhite et al. [19] directly measured contact angles of the snake skin and showed its hydrophobic properties. The mechanical properties of the skin have been investigated in great detail by Klein and Gorb [20]. They revealed a depth gradient in stiffness: the skin consists of a hard, robust, inflexible outer surface and softer, flexible inner layers [20]. The topography of the skin is well known for many snake species [4,6,8,11,18,21–34]. Some of the previous authors suggested that the microstructure of the ventral surface could be of high relevance for the snake locomotion [6,8,10–11,34].

**Figure 1:**
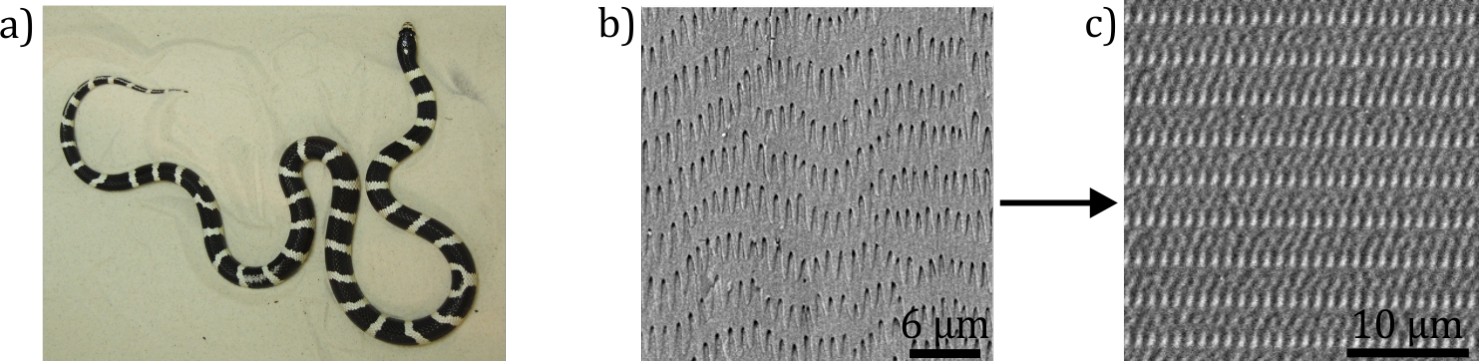
From the snake skin microstructure to the SIMPS. a) Photograph of the California King Snake (*Lampropeltis getula californiae*)*.* SEM-micrographs of the snake skin of a ventral scale (b) and the SIMPS made of epoxy resin (c).

*Lampropeltis getula californiae*, the California King Snake (Figure 1a) was recently chosen as biotribological model, because this snake lives in habitats with a relatively wide variety of substrates and therefore the skin modifications are presumably adapted for locomotion not just for one type of substrate. The microstructures on ventral scales are regular tooth-like shaped caudally-oriented (parallel to the body axis of the snake, see Figure 1b) with anisotropic frictional properties [10]. However, the complexity of the microstructure of this species is limited to the extent that it is suitable for transfer in artificial epoxy resin surfaces. Such artificial surfaces were used in this study for closer frictional characterization.

Due to the fact that controlled variation of the surface microstructure of the biological model is not possible, the investigation of the influence of the microstructure on the frictional properties is almost impossible, because of the absence of a control surface made of the same material. By using epoxy resin polymer surfaces for tribological investigations, we gained the opportunity to transfer the snake skin microstructure and other types of surface topographies into a well defined material by using two-step moulding technique [35]. Snake-inspired microstructured polymer surface (SIMPS) was developed in cooperation with the company Leonhard Kurz Group Stiftung & Co (Fürth, Germany). Its geometry is based on that of the ventral snake scales of *L. g. californiae* [10] (Figure 1c).

In the previous study, we have characterised frictional properties of shed snake skin from *L. g. californiae* and the influence of the stiffness of the underlying skin layers and the surface roughness of the substrate on the frictional coefficient [10]. In the present study, we used a similar experimental setup to characterise frictional properties of the SIMPS. Additionally, frictional properties of a broad variety of epoxy surfaces with different types and dimensions of the microstructure were characterised to understand the influence of two general tribological phenomena on friction: (1) molecular interaction depending on the real contact area between surfaces and (2) the interlocking of surface asperities of both contacting surfaces [36–38]. This approach of investigating the contribution of different geometries and dimensions of microstructures to the friction coefficient was chosen, because the complex phenomenon of friction cannot be reduced to a single mechanism: It is rather a result of various simultaneously acting mechanisms at different scales [39–41] and this approach opens up opportunities (i) to draw conclusions on the influence of the microstructure of the snake skin on frictional properties and thereby to extend the knowledge on specific surface modifications due to the legless locomotion of snakes and (ii) to evaluate which particular features (shape, dimension, orientation) of the snake skin are worth of mimicking for technological applications.

## Results

### Surface morphology

**Figure 2:**
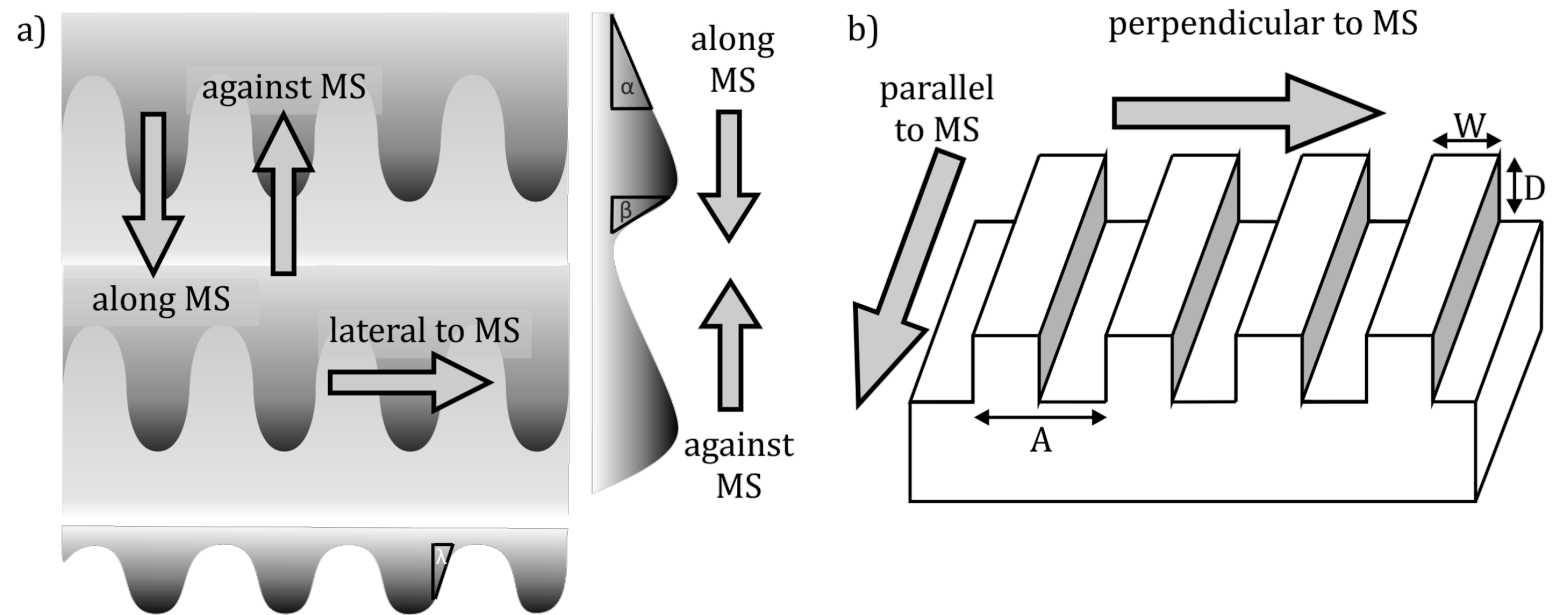
Scheme of the directionality of frictional measurements on polymer surfaces and its geometry. a) Directions of measurement relative to the topography of the SIMPS and angles of the microstructure depending on the measurement directions: α: along, β: against and λ: lateral to the microstructure. b) Diagram of the periodic groove-like profile. MS: microstructure, *A*: periodicity of the structure, *W*: width of the structure and *D*: depth of the grooves.

**Figure 3:**
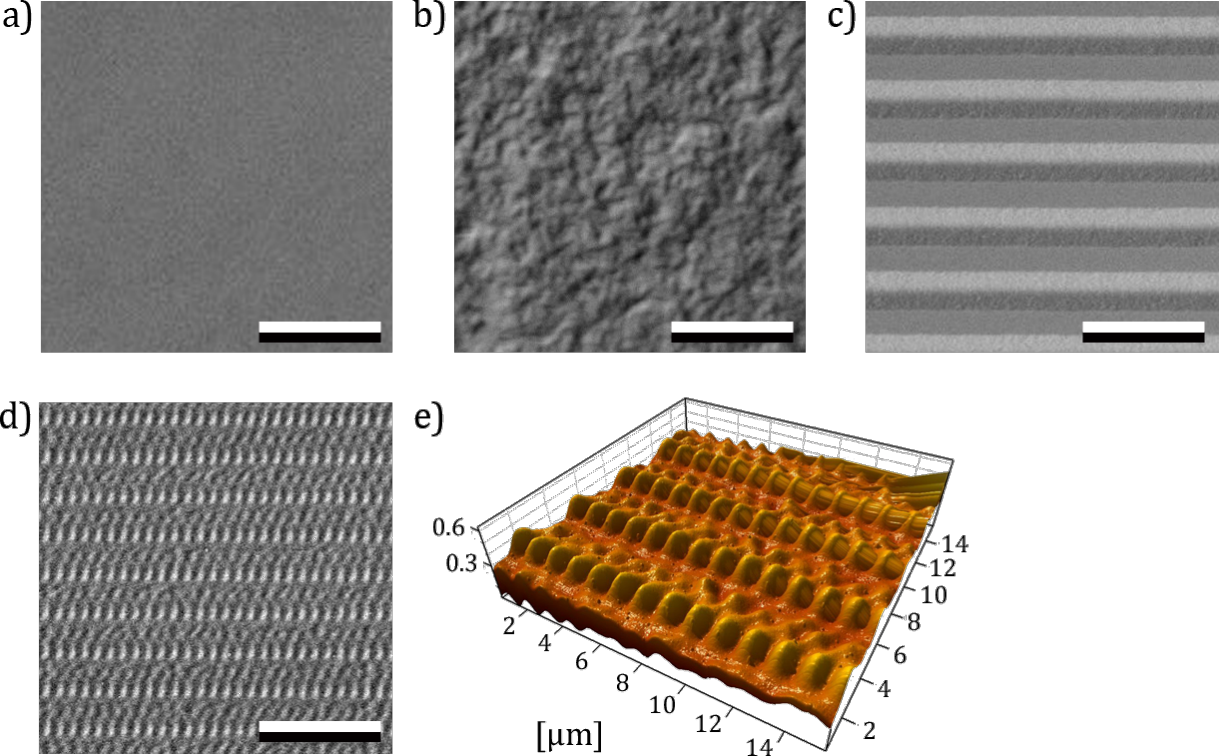
SEM (a–d) and AFM (e) micrographs of epoxy resin polymer moulds of different types of surfaces used in experiments. a) Smooth surface. b) Randomly rough surface with a grain size of 0.3 µm. c) Surface with periodically groove-like microstructures with a structural wavelength of 5 µm. d) SIMPS. Scale bars = 10 µm. e) 3D surface profile of the SIMPS.

The morphology of the SIMPS’ microstructure is much alike the biological model regarding the structural wavelength and the mean width of denticulations. The length of the denticulations on SIMPS is shorter than that of the snake surface, but the overall dimensions of the microstructures are comparable (Table 1). The geometrical anisotropy in form of slopes is present (Figure 2a). The angle in the direction of the measurement along the SIMPS’ microstructure is 47°, in the opposite direction 62°, and in lateral direction 55° (Table 2, Figure 2a).The quality of the moulded polymer surfaces was inspected by SEM and AFM (Figure 3). To ensure the absence of abrasion on the surface of the probe (a smooth glass ball), its surface was repeatedly examined by white light interferometer (data not shown).

**Table 1:** Dimensions of the microstructures of the ventral scales of the snake *L. g. californiae* and the SIMPS.

	structural wavelength of the microstructure [µm]	length of the denticulations [µm]	mean width of the denticulations [µm]

*L. g. californiae*	1.1 ± 0.1	2.5 ± 0.5	0.6 ± 0.1
SIMPS	1.2 ± 0.1	1.1 ± 0.2	0.7 ± 0.2

**Table 2:** Frictional coefficients (mean values and standard deviations) measured on SIMPS and angles of microstructure. Arrows show sliding directions of each individual measurement.

surface type		frictional coefficient, µ	angle

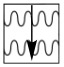	SIMPS - along the microstructure	0.165 ± 0.010	47°
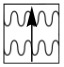	SIMPS - against the microstructure	0.245 ± 0.019	62°
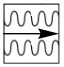	SIMPS - lateral to the microstructure	0.250 ± 0.018	55°

**Figure 4:**
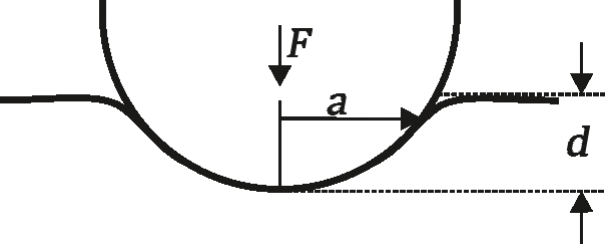
Contact area between sphere and flat elastic surface. *F*: Normal force. *a*: indentation radius. *d*: Indentation depth. Image modified after Popov [37].

As an indication for the maximum contact area occurring in our measurements we estimated the Hertzian contact area [42] of the glass sphere in contact with flat substrate according to the following parameters. The radius of the glass sphere was *R*_s_ = 0.5 mm. For the glass sphere, an elastic modulus of 70 GPa and a Poisson's ratio of 0.2 were assumed [43]. The elastic modulus of the epoxy resin was estimated to be 7 GPa and the Poisson's ratio to be 0.5 [43]. The geometric deformation between the sphere and flat surface under an applied normal force *F* was characterised by the indentation depth *d* and indentation radius *a* (Figure 4).

The contact radius *a* can be described by [37]:

[1]
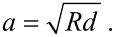


The relationship between applied force and geometrical deformation can be described by the following formula [37]:

[2]
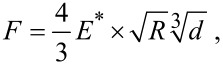


where

[3]
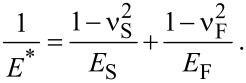


**Figure 5:**
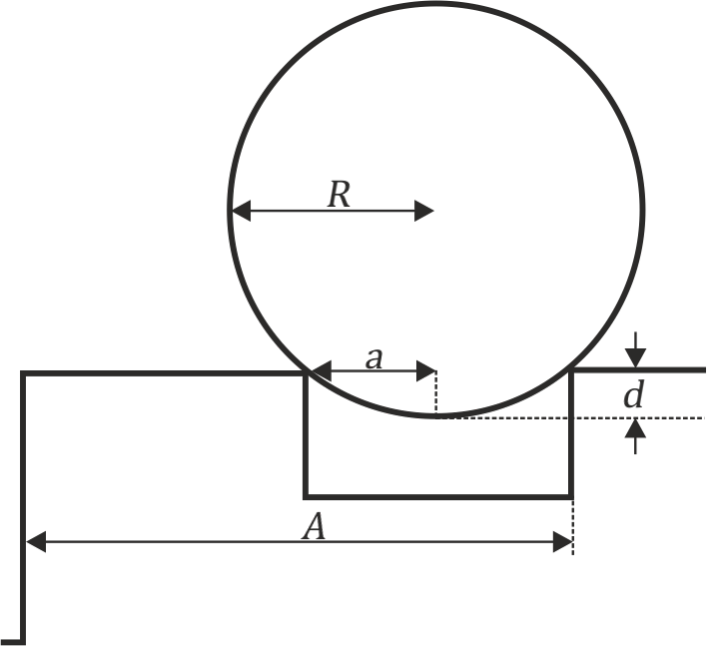
Geometric interaction between sphere and PGMS. *R*: sphere radius. *a*: indentation radius. *d*: indentation depth. The elastic response of the materials is excluded. Image modified after Sondhauß et al. [38].

The calculated contact area between the tribo-pair on smooth and flat surfaces was 40 µm^2^ corresponding to a contact radius of 3.5 µm and an indentation depth of 25 nm. The actual contact areas in case of the microstructured surfaces were indeed smaller. The following calculations are dealing with the geometric dimensions between the periodicity of the microstructures and the glass ball as counterpart, without applying a normal force (Figure 5). Detailed calculations are only possible for groove-like microstructure polymer surfaces (PGMS) due to the regular and well defined surface microstructures. Information on the exact geometry of PGMS topography is listed in Table 3. The measured details are described in Figure 2.

**Table 3:** Exact geometry of each PGMS pattern (mean values and standard deviations) measured by white-light interferometer. λ: pitch dimension, *A*: periodicity of the structure, *W*: width of the structure and *D*: depth of the grooves.

sample	*A* [µm]	*W* [µm]	*D* [µm]

PGMS - λ = 5 µm	5.3 ± 0.5	2.2 ± 0.1	2.2 ± 0.3
PGMS - λ = 25 µm	24.9 ± 1.8	13.1 ± 1.1	5.2 ± 0.4
PGMS - λ = 50 µm	50.0 ± 0.3	23.8 ± 2.3	17.7 ± 0.4
PGMS - λ = 100 µm	100.4 ± 1.9	49.4 ± 2.6	34.2 ± 1.2

The indentation depth *h* of the glass sphere was calculated according to the following formula modified after Sondhauß et al. [38]. With the indentation radius *a* and the periodicity of the structure *A*.

[4]
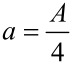


and

[5]
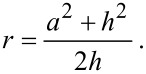


The calculated indentation depth of the glass ball into the microstructures without material deformation is listed in Table 4. It is necessary to emphasise that this theoretical indentation depth means the depth of penetration of the spherical cap just by geometry. The real indentation depth of the glass ball into the microstructures under a certain applied normal force is a combination of the material deformation and geometric conditions. Hence, this calculated penetration depth describes the minimal penetration depth between the tribo-pair.

**Table 4:** Calculated indentation of the glass ball into the PGMS depending on their pitch dimension. The calculated values, which lay beyond the spacial resolution of the system in normal direction (50 nm), are highlighted in bold.

	PGMS - 5 µm	PGMS - 25 µm	PGMS - 50 µm	PGMS - 100 µm

indentation depth [µm]	**0.002**	**0.039**	0.156	0.625

It is necessary to notice that the spacial resolution of the microtribometer for cantilever deflection in normal direction is too low to detect the deflection due to the penetration of the glass ball into microstructures of pitch dimensions of 5 µm and 25 µm (Table 4). Conclusions can be drawn on the interaction between the glass sphere and the geometries of the other microstructured surfaces due to the comparison of surface roughness of the investigated surfaces (Table 5). Referring to the threshold of detectable microstructure dimensions and the corresponding roughness value, it can be concluded that the interaction between the investigated surfaces (except for PGMS - 50 µm and PGMS - 100 µm) and the sphere is not exclusively caused by a vertical interlocking due to indenting into the microstructure.

**Table 5:** Surface roughness (*R*_a_) of all investigated polymer surfaces. λ: pitch dimension. SD: standard deviation. For PGMS, the surface roughness was measured perpendicular to the microstructure for each type of pitch dimension. In parallel direction to the PGMS microstructures, the roughness was averaged over all pitch dimensions.

sample			*R*_a_ ± SD [µm]

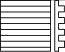	periodic groove-like microstructure	PGMS - λ = 5 µm	0.18 ± 0.022
	periodic groove-like microstructure	PGMS - λ = 25 µm	4.95 ± 0.369
	periodic groove-like microstructure	PGMS - λ = 50 µm	21.75 ± 0.262
	periodic groove-like microstructure	PGMS - λ = 100 µm	42.50 ± 1.465
	periodic groove-like microstructure	PGMS – on line	0.03 ± 0.005

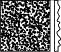	randomly rough surface	RRS - 0.3 µm	0.23 ± 0.004
	randomly rough surface	RRS - 1 µm	0.41 ± 0.013
	randomly rough surface	RRS - 3 µm	1.11 ± 0.106
	randomly rough surface	RRS - 9 µm	2.39 ± 0.072
	randomly rough surface	RRS - 12 µm	7.64 ± 0.127

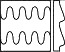	snake-inspired microstructured surface	SIMPS	0.10 ± 0.130

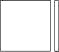	smooth surface	smooth surface	0.02 ± 0.007

### Frictional measurements

**Figure 6:**
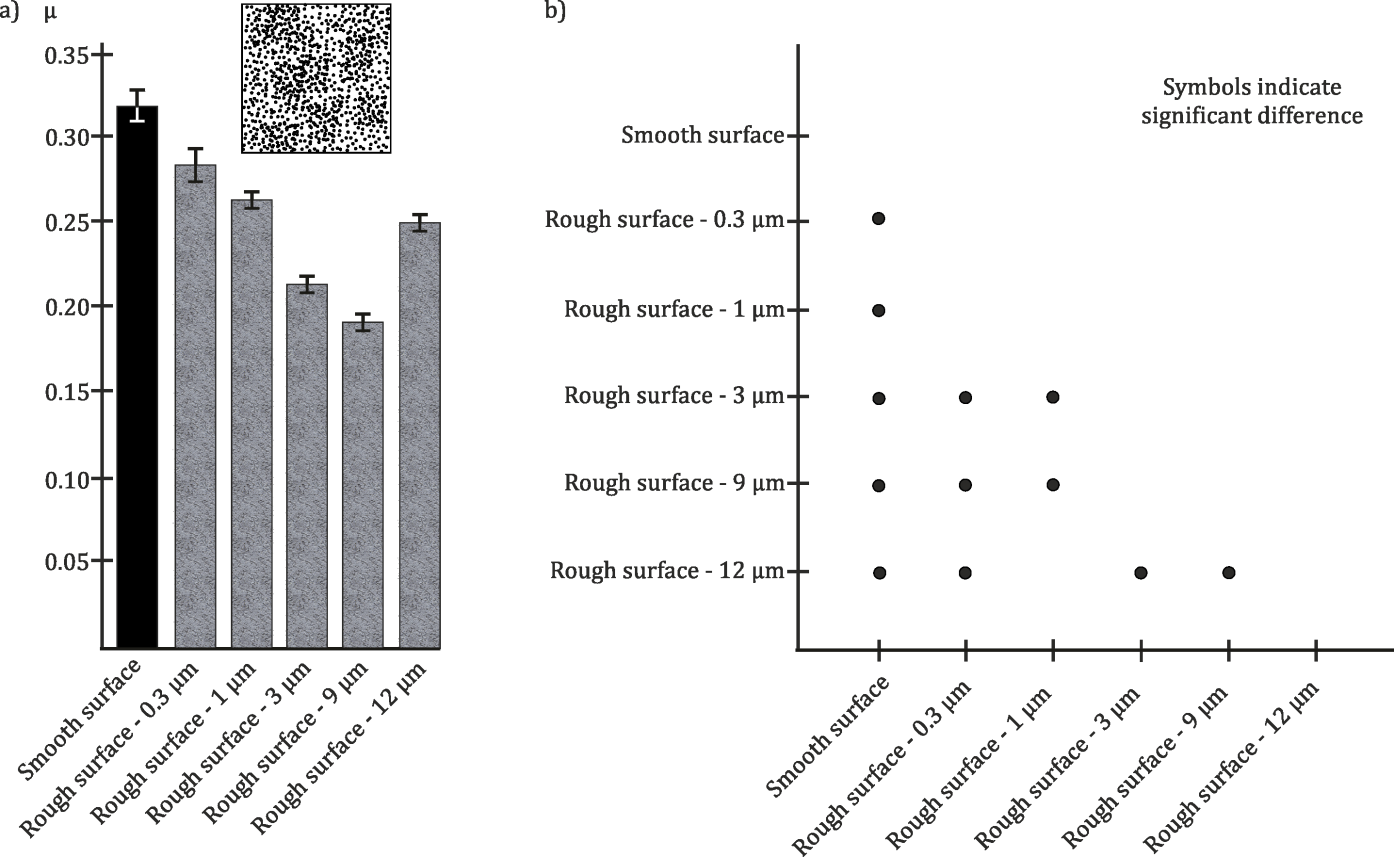
a) Results of tribological characterization of microstructured polymer surfaces in contact with a smooth glass ball. Average frictional coefficients (µ) and standard deviations are shown. Black column: smooth surface as reference. Gray columns: epoxy resin moulds of randomly rough surface with different grain size (*R*_a_: 0.3 µm, 1 µm, 3 µm, 9 µm, and 12 µm). b) Multiple comparison graph of the results presented in a). Dots indicate statistically significant differences between samples.

The microtribological measurements on the rough polymer surfaces (Figure 6) showed the lowest frictional coefficient (0.192 ± 0.007) at a grain size of 9 µm. Comparing this with the one measured on the smooth polymer surface (0.318 ± 0.024), a reduction in friction of about 40% was observed. The frictional coefficients decreased from the highest value on the smooth surface to the minimum at a specific grain size of 9 µm and further increased at a grain size of 12 µm. All frictional coefficients differed significantly from each other, except between surfaces with grain sizes of 0.3 µm/1 µm, 1 µm/12 µm, and 3 µm/9 µm (Figure 6b).

**Figure 7:**
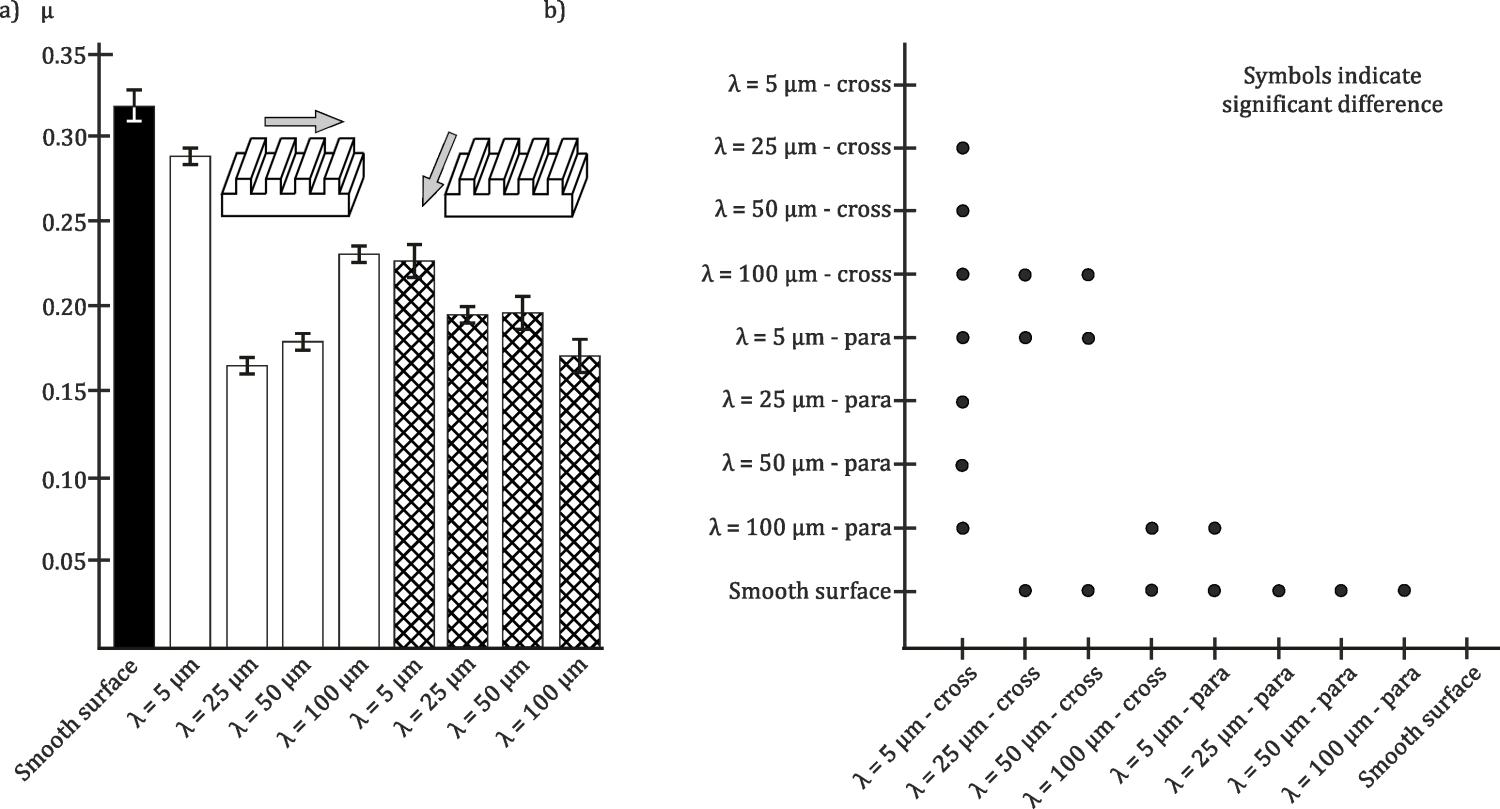
a) Results of tribological measurements of PGMS with different wavelengths (λ) in contact with a glass ball. The dimensions (structural wavelengths) of microstructures are 5 µm, 25 µm, 50 µm, and 100 µm, respectively. To investigate the influence of the geometry of the microstructure on frictional properties, measurements were performed in two perpendicular sliding directions. Black column: smooth surface as reference. White columns: sliding direction perpendicular to the groove pattern. Patterned columns: sliding direction parallel to the grooves. Average frictional coefficients (µ) and standard deviations are shown. b) Multiple comparison graph of the results presented in a). Dots indicate statistically significant differences between samples.

The frictional measurements on the periodical groove-like microstructure polymer surfaces (PGMS) were performed in two perpendicular sliding directions. The measurements perpendicular to the orientation of the microstructure with structural wavelength dimensions of 25 µm, 50 µm, and 100 µm revealed a lower frictional coefficient, if compared to the smooth polymer surface. The coefficient, measured for a structural wavelength of 5 µm, was very similar to the one of the smooth polymer surface. A minimum of friction was observed at a wavelength dimension of 25 µm. In this case, the frictional coefficient was 49% lower than that for the smooth surface (Figure 7).

If it is compared to the smooth surface, a reduction in friction of 44% was observed when measuring parallel to microstructure of the PGMS with a structural wavelength of 100 µm, but no minimum of frictional coefficient was detected within the parallel measurements (Figure 7). There was no statistically significant difference between the surfaces with different wavelengths. An interlocking effect, like the one detected in perpendicular direction, was not observed in parallel measurements. Nevertheless, any type of microstructure provided a statistically significant reduction in frictional coefficient, if compared with the smooth control surface (Figure 7b).

**Figure 8:**
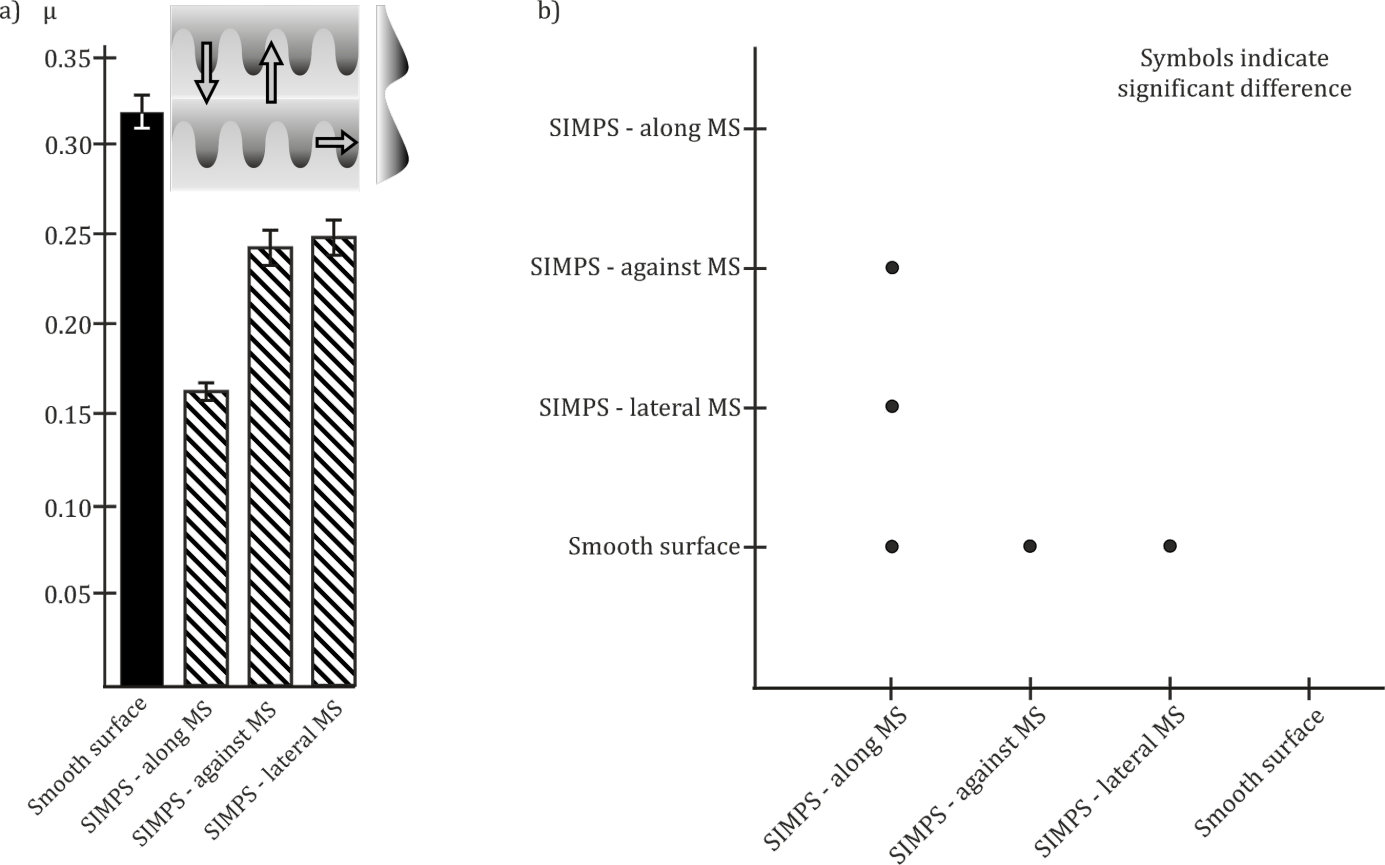
a) Results of tribological measurements of SIMPS in contact with a glass ball. Black column: smooth surface as reference. Hatched columns: SIMPS. To investigate the influence of the anisotropic geometry of the microstructure on frictional properties, measurements were performed in three different sliding directions. MS: microstructure. Average frictional coefficients (µ) and standard deviations are shown. b) Multiple comparison graph of the results presented in a). The dots indicate statistically significant differences between samples.

Frictional measurements on SIMPS showed anisotropic frictional properties and a reduction of the frictional coefficient of 48% measured along the microstructures, if it is compared to the smooth surface. There was significant difference between frictional coefficients on the smooth surface and the measurements against to the microstructure of the SIMPS and in the lateral directions (Figure 8). A statistically significant anisotropy was found between the measurement directions (i) “along” versus “against” the microstructure and (ii) “along” versus “lateral” to the microstructure orientation (frictional coefficient was reduced by 33% and 34%, respectively). All results of frictional measurements are listed in Table 6.

**Table 6:** Frictional coefficients (mean values and standard deviations) of all examined polymer surfaces. The arrows show the sliding directions of each individual measurement.

surface type		frictional coefficient, µ

smooth surface		0.318 ± 0.024
randomly rough surface - 0.3 µm		0.284 ± 0.027
randomly rough surface - 1 µm		0.264 ± 0.008
randomly rough surface - 3 µm		0.214 ± 0.011
randomly rough surface - 9 µm		0.192 ± 0.007
randomly rough surface - 12 µm		0.250 ± 0.013
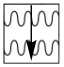	SIMPS - along the microstructure	0.165 ± 0.010
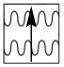	SIMPS - against the microstructure	0.245 ± 0.019
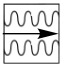	SIMPS - lateral to the microstructure	0.250 ± 0.018
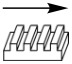	λ = 5 µm	0.290 ± 0.006
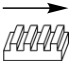	λ = 25 µm	0.167 ± 0.008
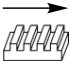	λ = 50 µm	0.181 ± 0.006
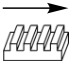	λ = 100 µm	0.232 ± 0.006
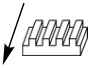	λ = 5 µm	0.228 ± 0.016
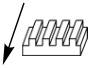	λ = 25 µm	0.196 ± 0.011
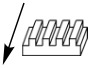	λ = 50 µm	0.198 ± 0.022
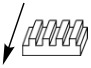	λ = 100 µm	0.172 ± 0.024

## Discussion

SIMPS with their anisotropic microstructure geometry exhibited anisotropic frictional properties similar to those of the biological model [5,8,10–11]. Additionally, SIMPS demonstrated a considerable reduction of the frictional coefficient, if compared to the same polymer with smooth surface.

Different kinds of microstructured surfaces with isotropic and anisotropic microstructure geometry of different dimensions were examined to gain a deeper understanding of how frictional properties are influenced by surface topography. The results obtained can be explained by mechanical interactions between surfaces at two scales: at a nano scale by the influence of the real contact area, and at a micro scale by an interlocking of the probe with the valleys of the structured counter surface. These findings indicate that the dimension of the best friction minimizing microstructure reduces the real contact area with the tribo-pair as far as possible without enabling mechanical interlocking. Nevertheless, as Baum et al. [44] have shown, frictional behaviour in respect to stick–slip behaviour is strongly influenced by the dimension of surface microstructures even when no mechanical interlocking occurs. One possible explanation for this phenomenon is that the microornamentation causes a critical stiction length, which leads to a periodical variation in real contact area between the tribo-pair causing friction-induced vibrations. Baum et al. [44] investigated this effect by fast Fourier transformation in detail and have shown that frictional behaviour is strongly influenced by real contact area, the possibility of mechanical interlocking, but beyond these parameters, also a critical stiction length due to microstructure dimension is of importance. Furthermore Sondhauß et al. [38] investigated the influence of microstructures in a smaller dimension on frictional properties. By using a friction force microscope, they have shown that the macroscopic interlocking of the probe tip and the surface microstructure leads to an increase of the frictional coefficient in a dry sliding system. This increase is caused by the interlocking, when the probe tip leaves the valleys of the microstructure and “climbs up” its edge. Hazel et al. [11] found a comparable situation for the skin surface of snakes. Sondhauß et al. [38] reported a 50% reduction in friction coefficient measured for the contact pair sphere–microstructure, when interlocking was not possible. A similar reduction in friction was also found in our experiments for frictional measurements on PGMS in perpendicular direction to the microstructure at a dimension of 25 µm. Sondhauß et al. [38] and Baum et al. [44] concluded that the frictional response is dominated by the geometry of the tribo-pair. Based on these assumptions they stated that moderate modification of surface roughness can improve the tribological performance of meso scale contacts.

The influence of surface roughness and thereby the contact area between the tribo-pair have been controversially discussed in literature for a long period of time. Based on Amontons' friction law [36–37,39–41,45], it is stated, that friction is proportional to the normal force and independent of contact area, thereby the influence of the roughness of the friction is minor, as long, as the roughness is low. This assumption can be confirmed for macroscopic frictional contacts [36–37,39,41,45]. Later it was stated that the real contact area between a tribo-pair is formed by multi-asperity contacts that enable a molecular interaction of the surface molecules and constitutes only a small percentage of the macroscopic contact area [36–37,39,45–46]. These close contacts are called junctions and the sum of all junctions forms the real contact area. This theory also explains the correlation between frictional coefficient and applied normal force, because an increase of normal force leads to an increase of the area of each asperity in contact and thereby to an increase of the molecular interaction between the surfaces. The degree to which the real contact area is influence by the load force depends strongly on material properties [41,46–48]. These properties are also affected by the surface geometry [49]. There are numerous experimental studies on the roughness effect on friction of technical surfaces. Etsion [50] and Kovalchenko et al. [51] investigated the role of the microstructuring with regular circular holes in micrometer dimensions on friction and concluded that artificial microstructuring of surfaces is a possibility to enhance the control of frictional system behaviour. They showed that a higher density of dimples lead to a stronger abrasive wear on the tribo-pair. Nevertheless by such kind of surface modification, they were able to reduce the frictional coefficient. A comparison of these finding with the results of our study is only possible in a limited way, because they used for their investigation a different machinery type (pin-on-disk friction machine), the micro dimples were much bigger than our microstructures (diameter: 58–78 µm) and they investigated a lubricated system. The observation of the effect that a specific surface roughness leads to the enhancement of the friction coefficient is congruent with ours, nevertheless it is necessary to notice that their ratio between spherical probe and line width (sphere diameter: 2.3 µm and 7.9 µm and line width: 0.5 µm to 3.5 µm) is different from our sphere/line width ratio (sphere diameter: 1 mm, line width: 5 µm to 100 µm).

Marchetto et al. [52] reported a reduction of the frictional coefficient measured perpendicular to linearly-grooved microstructured surfaces to about 36% in comparison to a smooth surface. Frictional measurements in two different directions (parallel and perpendicular to the line-grooved microstructure) showed equivalent values, meaning there were no anisotropic frictional properties. The reduction in friction is in accordance to our findings, but it is necessary to mention, that the contact geometry in Marchetto et al. [52] is different to our experimental setup, because they used a cut-off AFM cantilever tip. One can assume that a decrease in frictional coefficient is due to the reduction of adhesive components of the frictional mechanisms [51–52]. Another approach to explain the reduction in frictional coefficient on many microstructured surfaces could be the possibility of trapping of loose wear particles within the microstructures and thereby the avoidance of further surface ploughing by these wear particles [38,48,50,53].

The gap between biologically and artificially microstructured surfaces can be closed by interpreting the microstructure of the SIMPS as lines and spaces, in which the lines are periodically interrupted by the elevated tips (denticulations). The SIMPS microstructure is based on shapes and dimensions of the microornamentation of the biological model, the ventral scales of the snake *L. g. californiae*. For the biological model, it was previously assumed, that the caudal tips of denticulations are elevated, so the snake can generate propulsion due to the interlocking of its microstructure with surface asperities. The results of the study of the snake skin’s microstructure by using atomic force microscopy (AFM) and confocal laser scanning microscopy (CLSM) showed that the anisotropic geometry of the surface structure is not dominated by the elevation of the caudal tips of the denticulations as previously found on *Boa constrictor* skin [11], but rather by depressions located cranially between the denticulations [10]. The dimension of the microstructure on the SIMPS is quite similar to those of the ventral scales of the investigated snake species, but there is a difference in topography: In SIMPS, the caudal tips are slightly elevated, whereas in *L. g. californiae* they are not.

In the present study, SIMPS showed a similar level of frictional anisotropy as the uncushioned snake skin [10], enforcing the hypothesis that the snake-like surface microstructure contributes to the frictional anisotropy. Previous studies have demonstrated that frictional coefficients can be controlled by using different kinds of microstructures on technical surfaces [38,50–52,54] the specific geometry of the microstructure was investigated by Wang et al. [55], Galda et al. [56], Yu et al. [57], Prodanov et al. [58], Gachot et al. [59] and Filippov and Gorb [60]. The role of the specific geometry of the microstructure and its angle in relation to the direction of sliding was in focus of Abdel-Aal [61]. Anisotropic frictional properties, shown in the present paper, can be explained by mechanical interlocking of multiple micro asperities and by the variation of the contact area depending on the angle of the microstructure. This statement is strengthened by the following observation. The angle along the microstructure is 25% smaller than in the opposite and 17% smaller than in lateral direction. The frictional measurements on SIMPS showed a similar distribution of the frictional coefficients measured along and against the microstructure, but not in lateral direction compared to both other directions (Table 6). Despite the fact that frictional anisotropy is not completely congruent to the angle distribution, it can be derived, that the slope of surface topography influences frictional properties, as proposed by, e.g., Abdel-Aal [61], Persson [36] and Popov [37].

Our experiments reveal an influence of the surface roughness on the friction of dry polymeric systems in contact with smooth surface: In general, we recorded lower values on rough surfaces, if compared to the smooth reference surface. It can be primarily explained by the lower real contact area between the tribo-pair. The data on surfaces with different roughness show a decrease of frictional coefficient with growing grain size until a minimum friction is reached at 9 µm. The observed effect of a decreasing frictional coefficient, µ, with increasing roughness is reversed at 12 µm grain size presumably by another type of tribo-pair interaction. While the decreasing part of the curve can be explained by the reduction of the contact area of the tribo-pair, the increasing part is rather due to the interlocking between the sphere and large surface asperities. The indentation radius of the glass ball on the polymer surface was 3.5 µm, which should be sufficient for interlocking with a rough surface having a grain size of 12 µm and a roughness (*R*_a_) of 7.64 µm. The interlocking effect is presumably rather strong here and obviously reverses the friction minimizing effect due to the decreased contact area of rough surfaces. These effects have been described by various authors investigating frictional behaviour of technical surfaces [38,52,54] or biological surfaces [6,11].

In order to understand the influence of the periodic anisotropic surface roughness, further frictional measurements on periodically groove-like microstructured polymer surfaces were performed here. Frictional measurements perpendicular to the periodical groove-like microstructure showed a maximal reduction of µ to 49% at a structural wavelength of 25 µm, if compared to the smooth reference. The results obtained on PGMS with a structural wavelength of 5 µm were similar to those on the smooth surface. We assume that frictional behaviour of the latter contact pair can be explained by the relatively big real contact area. At λ = 25 µm, the contact area is presumably much smaller than at λ = 5 µm. Measurements on PGMS with larger λ (50 and 100 µm) showed an increase of the frictional coefficient. Similar to the experiments on polymer surfaces with different roughness (see above), these differences in frictional behaviour result from the interplay between two effects: (1) the decreasing of the real contact area at small λ and (2) the increasing of the mechanical interlocking between the sphere and surface topography at large λ (Figure 7).

Measurements in the direction parallel to the microstructure excluded the possibility of interlocking and were dominated by the real contact area effect, which was rather constant for samples with different wavelengths. The data do not show statistically significant differences of µ with an increasing wavelength. By comparing the results obtained in measurements perpendicular and parallel to the PGMS, it is possible to consider both physical phenomena which influence the friction in a dry polymeric system: the real contact area and mechanical interlocking between the tribo-pair. By comparison of the results obtained in both directions within the different wavelength of microstructures, a significant difference was detected only for λ = 5 µm. It can be deduced that interlocking with the microstructure occurs, but its effect on frictional coefficient is minor (no significant difference, but variations in absolute frictional coefficient). In a comparable experimental setup, Yu and Wang [57] investigated whether anisotropic frictional properties do change with topographic parameters, and, thereby, whether the modification of microstructures is a way to modulate friction. They used groove-textured surfaces and performed frictional measurement on two different combinations of sphere and microstructure dimension. For the combination of a tungsten carbide sphere (diameter = 800 µm) and a microstructured silica surface (λ = 278 nm) the frictional coefficient parallel to the microstructure was higher, than in the perpendicular direction. The second investigated frictional pair was a steel ball (diameter = 800 µm) in contact with a microstructured tungsten carbide surface (λ = 220 µm). For this combination, the frictional coefficients in both directions were similar, but in the perpendicular direction they were slightly higher. Similar experiments were done by Marchetto et al. [38], during which they also observed the absence of anisotropy on periodically groove-like microstructure, but the influence of different dimensions of microstructures on this effect was not investigated.

Our above experiments have demonstrated dimensional effects of microstructure on friction and strong effect of their shape. There is a trend for the reduction of friction with increasing dimensions of the microstructure until the interlocking effects start to occur. It has been previously shown that the attachment ability of insects [62–65] and geckos [66] is strongly dependent on the surface roughness. Yu et al. [67] demonstrated that surface roughness also strongly affects the performance of gecko-inspired adhesives. All these authors have shown that there is a critical roughness, on which the attachment ability (both adhesion and friction) is strongly reduced. The interlocking effect, contributing to the friction increase, was observed taking place at larger dimensions of the microstructure, as shown for frictional measurements perpendicular to the PGMS with a structural wave length of both 50 µm and 100 µm.

A global comparison of all samples studied shows that minimal frictional coefficients were obtained (1) perpendicular to the PGMS with a structural wavelength of 25 µm, (2) on a surface grain size of 9 µm, (3) on SIMPS measured along the microstructure and (4) parallel to the PGMS with a structural wavelength of 100 µm. What do these microstructured surfaces have in common? Most of them (1)–(3) possess a meso scale surface roughness, resulting in the best compromise between a reduction of the real contact area (and therefore reduction of adhesion) and the prevention of the interlocking effect. The only exception (4) was the measurement parallel to the PGMS with a structural wavelength of 100 µm, because here geometrical interlocking was not possible. In this case, it would be most interesting to investigate in the future, if a further minimization of the frictional coefficient would be possible, if the periodicity of the microstructures would be so wide that the sphere would only be in contact in between two lines (something similar to the effect of micro rails).

We showed that the reduction of the real contact area leads to a minimization of the frictional coefficient, but this possibility of optimisation is limited by an interlocking of surface structures. This conclusion is in accordance to Marchetto et al. [52] and Sondhauß et al. [38]. Because the frictional optimisation in a dry sliding frictional system strongly depends on the dimension and shape of surface asperities of the tribo-pair, an optimisation of the frictional surfaces by surface texturing must be done individually for each type of friction contact pair, as previously proposed [50]. However, the present work provides some ideas for the implementation of surface microstructures of particular dimensions and shape for the reduction of friction of polymeric systems. Additionally, we have clearly shown that the use of inspiration from sliding biological tribosystems, such as snake skin, may provide a short cut to development of novel tribologically optimised polymer surfaces. However, in long term experiments, the geometry of the surface microstructure can undergo some evolution due to abrasive wear, and therefore enhanced frictional properties can change due to the degeneration of surfaces [50–51]. However, the wear of the SIMPS was not in the focus of this study, but should be done in future investigations.

## Experimental

### Microstructured surfaces

Friction measurements were performed on four different types of microstructured surfaces. The first type of surfaces (control) originated from the mould of a smooth glass surface (Figure 3a). The second type of surfaces originated from moulds of polishing paper (FibrMet Discs, Buehler GmbH, Düsseldorf, Germany) with different grain sizes (*R_a_*: 0.3 µm, 1 µm, 3 µm, 9 µm, and 12 µm) (Figure 3b). The master for the third type of surfaces was produced from zirconium oxide surface microstructured by femtosecond laser ablation. Structuring was performed with a commercially available amplified Ti:Sapphire femtosecond laser system (Femtopower Compact Pro, Femtolasers GmbH, Austria). The systems delivers sub-30-fs pulses at a central wavelength of 800 nm with a pulse energy of up to 1 mJ, and a repetition rate of 1 kHz. An x–y motorized translation stage (Physik Instrumente GmbH, Germany) was used for sample positioning and translation. A computer controlled LCD element was used for setting the laser pulse energy. It features periodic groove-like microstructures (PGM) with different structural wavelengths of 5 µm, 25 µm, 50 µm, and 100 µm (Figure 3c). The fourth type of surfaces was inspired by the microornamentation of the ventral scales of the snake *L. g. californiae* (snake-inspired microstructured polymer surface, SIMPS) (Figure 3d). The masters were produced by the Leonhard Kurz Group Stiftung & Co. (Fürth, Germany) by using e-beam greytone lithography with a negative photoresist. Afterwards nickel copies were manufactured through an electroplating process.

Replication of the microstructures was performed by using a two-step moulding technique according to Gorb [35]. The surface that ought to be replicated was used as a master and, in the first step, was covered with fluid polyvinylsiloxane (PVS), a two-component silicone, which polymerizes within minutes at room temperature (Coltène President light body, Coltène Whaledent Dentalvertriebs Ltd., Constance, Germany). The obtained negative cast was filled out by Spurr’s low-viscosity resin [68]. The polymerization of the resin took place overnight at 70° C. The resin (Polysciences Inc., Eppelheim, Germany) consists of nonenyl succinic anhydride (NSA) (61.3%), 3,4-epoxycyclohexylmethyl-3,4-epoxycyclohexylcarboxylate (ERL 4221) (23.6%), diglycidyl ether of polypropyleneglycol (D.E.R. 736) (14.2%) and *N*,*N*-dimethylaminoethanol (DMAE) (0.9%).

### Visualization

The microstructure of the obtained polymer surfaces were visualized by means of a scanning electron microscope (SEM). The SEM investigations were performed with a Hitachi S-4800 (Hitachi High-Technologies Corporation, Tokyo, Japan) at an acceleration voltage of 2–3 kV and a Hitachi TM3000 (Hitachi High-Technologies Corporation, Tokyo, Japan) at an acceleration voltage of 5 kV. Prior to visualization, the material was fixed to the aluminium stub with a carbon-bearing adhesive pad and sputter-coated with a 20 nm thick gold-palladium (4:1) layer by using a high vacuum sputter coater Leica EM SCD500 (Leica Microsystems GmbH, Wetzlar, Germany). Additionally, for quick 3D surface observations a white-light interferometer (New View 6000, ZygoLOT, Darmstadt, Germany) without the sputter coating was used.

As described in [44], the detailed characterization of the surface topography was performed by a NanoWizard^®^ atomic force microscope (JPK Instruments), mounted on an inverted light microscope (Zeiss Axiovert 135, Carl Zeiss MicroImaging GmbH). The SIMPS were imaged by using the intermittent contact mode of the AFM. The error channel (also known as the amplitude channel) visualizes the change in damping of the cantilever amplitude while scanning the surface. Only images obtained with the error channel are shown, because this visualization method is helpful to gain a more vivid imaging of the surface topography. Scans were carried out at a 1 Hz scan rate and a resolution of 1024 × 1024 pixels with an intermittent contact mode cantilever (*c* = 50 N·m^−1^, NST-NCHF, Nascatec GmbH, Stuttgart, Germany), at ambient conditions (room temperature 24° C, relative humidity 41%). NanoWizard® SPM software 3.3.23 (JPK Instruments) was used to obtain AFM images and NanoWizard® image processing software 3.3.25 was applied to extract 3D surface profiles. The variables of microstructured surfaces were measured from digital images by means of the image analysis software SigmaScanPro 5.0 (SPSS Inc., Chicago, USA).

### Frictional measurements

Frictional coefficients, µ, were defined according to the Amontons' friction law: µ = *F*_t_*/F*_n_ (*F*_t_: tangential force; *F*_n_: normal force). The experimental parameters of the frictional measurements were chosen as described before [10], except for the usage of a rough glass ball. The E-moduli of the polymerized Spurr resin and the glass ball were 7 GPa and 70 GPa, respectively [43]. The maximum contact area between the glass ball and a smooth polymer under the given load was estimated according to the Hertz model [42].

To characterise frictional properties of the SIMPS, the measurements were performed in three different sliding directions: along the anisotropic microstructure (i), against the anisotropic microstructure (ii), and in the lateral direction, perpendicular to both other directions (iii) (Figure 2). The frictional properties of the periodic groove-like patterned surface (PGMS) were characterised in two different directions only: parallel to the microstructure (i) and perpendicular to the microstructure (ii) (Figure 2b).

Individual measurements were repeated 15 times on each micro patterned surface and on the smooth reference surface. The other surfaces were tested five times each. Each measurement was performed on a new area of the surface to minimize the influence of abrasion. Obtained data were statistically analysed with SigmaPlot 11.0 software (SPSS Inc., Chicago, USA). Kruskal–Wallis one way ANOVAs followed by Holm–Sidak tests with a significance level of *p* < 0.05 were performed.
